# The effectiveness of symptom-oriented mind mapping combined with problem-based learning in critical care clerkships: a randomized controlled trial

**DOI:** 10.3389/fpubh.2025.1682687

**Published:** 2025-10-17

**Authors:** Tian Gao, Xuewei Zhao, Boheng Wang, Ling Wang, Yuan Mao, Dan Wang, Zhengyao Han, Vakkas Qureshi, Xueping Xu, Li Qian, Can Yang, Jie Yin, Runkai Shao, Wei Wang, Xilan Yang

**Affiliations:** ^1^The Fourth Affiliated Hospital of Nanjing Medical University, Nanjing, China; ^2^Nanjing Medical University, Nanjing, Jiangsu Province, China

**Keywords:** problem-based learning, mind mapping, critical care medicine, clinical clerkship, clinical reasoning

## Abstract

**Objective:**

This study evaluated the efficacy of Symptom-Oriented Mind Mapping combined with Problem-Based Learning (SOM-PBL) in intensive care unit (ICU) clinical clerkships, assessing its impact on medical students’ knowledge integration, clinical decision-making efficiency, and procedural skills, thereby providing theoretical and practical insights for optimizing critical care medical education.

**Methods:**

A prospective randomized controlled trial enrolled 160 fifth-year medical students during ICU clerkships at the Fourth Affiliated Hospital of Nanjing Medical University (January 2023–December 2024). Participants were randomly assigned to the SOM-PBL group (*n* = 80) or the control group (*n* = 80) using stratified randomization based on academic major and pre-clerkship academic performance. All students subsequently completed a four-week ICU clerkship using their assigned instructional method (SOM-PBL or traditional teaching), after which they underwent comprehensive assessments in theoretical knowledge, procedural skills, clinical reasoning, and educational satisfaction.

**Results:**

The SOM-PBL group achieved significantly higher median knowledge theoretical scores [median (IQR): 77 (73, 80) vs. 74 (72, 77); *p* < 0.05] and superior clinical reasoning performance [median (IQR): 88 (85, 93) vs. 87 (84, 89.75); *p* < 0.05]. No significant intergroup difference was observed in procedural skills performance [median (IQR): 92 (90, 94) for both groups; *p* = 0.938]. SOM-PBL showed significant strengths in monitoring technology/device application, pathophysiology/disease recognition, and diagnostic cognitive rigor (all *p* < 0.05). Over 80% of SOM-PBL students rated ≥90% of survey items as 4 or 5 on a 5-point Likert scale, indicating high satisfaction with learning efficiency, engagement, clinical reasoning enhancement, and course quality.

**Conclusion:**

The study demonstrated that the SOM-PBL approach significantly enhanced medical students’ knowledge integration and clinical decision-making efficiency during ICU clerkships. However, no significant improvement was observed in procedural skills. These findings offer both theoretical and practical value for innovating critical care medical education by effectively addressing knowledge fragmentation and bridging the theory-practice gaps.

## 1 Introduction

Critical care medicine, as a highly integrated interdisciplinary field, is fundamentally characterized by three core dimensions: multidisciplinary knowledge integration, analysis of complex pathophysiological mechanisms, and time-sensitive clinical decision-making (1). Critically ill patients frequently present with multisystem pathophysiological interplay, necessitating convergent expertise from internal medicine, surgery, radiology, and allied disciplines, alongside dynamic assessment and targeted interventions (2, 3). Nevertheless, contemporary critical care education systems face persistent challenges: traditional lecture-based learning perpetuates compartmentalized knowledge fragmentation due to disciplinary silos, while clinical clerkship rotations suffer from time-constrained training cycles, limited case exposure, and a lack of standardized protocols. Consequently, learners often exhibit deficiencies in integrative decision-making competence when managing complex clinical scenarios (4).

Since its systematic implementation at McMaster University School of Medicine in the 1960s, Problem-Based Learning (PBL) has evolved into a transformative paradigm in global medical education (5). This constructivist approach emphasizes scaffolded knowledge construction through socially interactive engagement within authentic problem scenarios, effecting a paradigm shift from passive didactic instruction (6). In critical care medicine, PBL cultivates evidence-based clinical reasoning and time-sensitive decision-making competencies by simulating clinical scenarios that guide learners through a cognitive sequence: problem identification, knowledge retrieval, collaborative decision-making, and reflective iteration (7–11). Empirical studies demonstrate that PBL cohorts achieve significantly superior performance in basic science examinations, particularly in knowledge perception, subject-specific retention, motivation, and peer-faculty communication (12). Furthermore, Bai et al. showed that an “atypical cases + PBL” approach substantially augments knowledge transfer competency, enhancing students’ capacity to identify non-classical pathological manifestations in clinical contexts (13). Thus, PBL epitomizes a core pedagogical strategy in modern medical education, reflecting a global consensus toward cultivating clinical meta-competence (14).

Mind mapping, originally developed by Tony Buzan in the 1970s, leverages bilateral hemispheric processing in neurocognition: left-lateralized logical analysis synergizes with right-dominant visuospatial integration, enabling dynamic knowledge association through dendritic frameworks (15). In critically ill patients, deceptively benign initial symptoms may indicate severe underlying pathologies, posing diagnostic challenges for junior clinicians. Empirical evidence confirms that mind mapping reduces cognitive load and enhances information processing depth through visual tools (e.g., chromatic coding, symbolic icons) (16, 17). Its implementation in nursing education significantly improved knowledge of pressure injury prevention and reduced incidence rates in critically ill patients. The integration of symptom-oriented mind mapping with PBL constitutes a convergent progression beyond additive combination. This synergy establishes a self-reinforcing learning cycle—problem-driven contextualization (PBL), knowledge structuring (Mind Mapping), and practice-based validation—where PBL anchors clinical scenarios while mind mapping deconstructs complexity into actionable subtasks, ensuring fidelity to learning objectives (18). For instance, in dental pedagogy, this multimodal integration enhanced motivation, knowledge retention, interdisciplinary connections, and collaborative competencies, with higher mentor competence ratings than PBL alone (19). Mind mapping reinforces analytical cognition through pathophysiological deconstruction, whereas PBL drives evidence-based decision-making via clinical paradoxes (20, 21). The synergistic integration of these pedagogies enables comprehensive trainee development across the clinical continuum—from disease onset and pathophysiological progression to differential diagnosis and management. This study aims to evaluate the efficacy of SOM-PBL in iICU clinical clerkships, assessing its impact on medical students’ knowledge integration, clinical decision-making efficiency, and procedural skills. The findings provide theoretical and practical insights for optimizing critical care medical education.

## 2 Materials and methods

### 2.1 Study design and setting

To provide context for international readers, the structure of medical education in this study is briefly outlined. The undergraduate medical program in China is typically a five-year curriculum, culminating in the award of a Bachelor of Medicine, Bachelor of Surgery (MBBS) degree. The ICU clerkship described in this study is a mandatory clinical rotation undertaken in the final (fifth) year. Prior to this rotation, students have typically completed core clinical clerkships in internal medicine, surgery, gynecology, pediatrics, and emergency medicine, which provide foundational knowledge in history-taking, physical examination, and common disease management. Upon entering the ICU rotation, students are expected to be competent in performing basic clinical skills, understanding fundamental disease pathophysiology, and interpreting common laboratory and imaging results. However, they are not expected to be proficient in the synthesis of complex, multi-system critical illness or the independent operation of advanced life-support technologies, which are the primary learning objectives of the ICU clerkship itself.

This prospective randomized controlled trial was conducted in the intensive care unit (ICU) of The Fourth Affiliated Hospital of Nanjing Medical University, a tertiary teaching hospital, between January 2023 and December 2024. The study protocol was approved by the Institutional Review Board of the hospital (approval number: 20231122-k164).

#### 2.1.1 Participants and sampling

The study population consisted of all fifth-year medical students undertaking their mandatory ICU clerkship during the study period. A consecutive sampling approach was used to invite all eligible students to participate. Participants were stratified based on academic major and pre-clerkship academic performance to ensure balanced distribution of these potential confounding factors across study groups10.

Inclusion criteria were: (1) fifth-year medical students; (2) undergoing ICU clerkship during the study period; and (3) provision of written informed consent.

Exclusion criteria were: (1) prior formal ICU training exceeding 2 weeks; (2) unwillingness to participate in the study; and (3) absence for more than 3 days during the clerkship period.

#### 2.1.2 Recruitment, enrollment, and ethical considerations

All eligible students were invited to participate through an orientation session conducted at the beginning of their ICU rotation. The study coordinator explained the study purpose, procedures, potential benefits and risks, and emphasized that participation would not affect their academic evaluation. Written informed consent was obtained from all participants before any study procedures were initiated. The study was conducted in accordance with the ethical principles of the Declaration of Helsinki.

#### 2.1.3 Randomization and blinding

We performed stratified block randomization based on two key prognostic factors: academic major and pre-clerkship academic performance (dichotomized as higher or lower than the median score). Within each unique stratum defined by the combination of major and performance level, participants were randomly allocated to either the SOM-PBL group or the control group using a computer-generated random sequence with varying block sizes (4 and 6) created in SPSS software (version 26.0, IBM Corp., United States) by an independent biostatistician.

Group assignments were concealed using sequentially numbered, opaque, sealed envelopes (SNOSE). After obtaining written informed consent, the research coordinator opened the next sequentially numbered envelope in the participant’s presence to reveal group assignment.

Due to the nature of the educational intervention, participants and teaching facilitators were not blinded to group assignment. However, outcome assessors (intensivists evaluating clinical reasoning and procedural skills) and data analysts remained blinded to group allocation throughout the study to minimize assessment and analytic bias.

#### 2.1.4 Interventions

Both groups completed a four-week supervised ICU clerkship with identical clinical exposure and content coverage, differing only in instructional methodology.

SOM-PBL Group: The intervention group received Symptom-Oriented Mind Mapping combined with Problem-Based Learning. The instructional approach included: (1) pre-designed structured templates with central symptoms (e.g., dyspnea) provided through MindManager software; (2) twice-weekly 90-min PBL sessions where student teams collaboratively expanded mind maps detailing etiological differentials, pathophysiological pathways, and evidence-based treatments; (3) authentic critical care cases (e.g., severe pneumonia with MODS) presented in scaffolded problem sequences progressing from symptom recognition to pathophysiological analysis and evidence-based decision-making; and (4) post-session submission of finalized mind maps for formative assessment with written feedback provided within 72 h.

Control Group: The control group received traditional lecture-based learning, which represented the conventional pedagogical approach in Chinese medical education. This instructor-centered method involved structured lectures and case presentations covering identical core topics—diseases, symptoms, and procedures—as those addressed in the SOM-PBL group.

### 2.2 Outcome measures and data collection

Data were collected at the end of the 4-week training period by blinded assessors using standardized instruments.

#### 2.2.1 Primary endpoints

**Theoretical knowledge** was assessed through a standardized written examination comprising multiple-choice questions (60–70%), fill-in-the-blank items (20–30%), and case analyses (10–20%). This exam evaluated core competencies in fundamental theories, pathophysiology, critical care monitoring technologies, emergency interventions, and common critical illnesses.

**Procedural skills performance** was assessed in a simulated setting using Objective Structured Clinical Examination (OSCE) stations. Participants performed essential critical care procedures, including thoracentesis, paracentesis, lumbar puncture, bone marrow aspiration, endotracheal intubation, central venous catheterization, and ARDS ventilator parameter optimization. Performance was evaluated with a standardized OSCE assessment form focusing on procedural standardization, aseptic technique, complication management, and emergency response.

#### 2.2.2 Secondary endpoints

**Clinical reasoning proficiency** was assessed independently by two board-certified intensivists (each with ≥5 years of specialization) using a modified Mini-CEX (Clinical Evaluation Exercise) form. This tool evaluated competencies in integrative history-taking, diagnostic reasoning, and time-sensitive decision-making.

#### 2.2.3 Assessment criteria

All procedural skills and clinical reasoning assessments were evaluated by two independent, blinded intensivists, each with over 5 years of specialization, to minimize bias.

Procedural skills were assessed across three primary domains (Aseptic Technique, Procedural Protocol, and Management of Adverse Events). Each domain was scored on a 0–10 scale. The total score was calculated by summing the scores of the three domains (maximum 30 points) and then scaled to 100 for consistency.

Clinical reasoning proficiency was assessed using a modified Mini-CEX form across three core competencies (Integrative Clinical History Analysis, Diagnostic Cognitive Rigor, and Time-Critical Decision Determinants). Each competency was rated on a 1–10 scale (1 = unsatisfactory, 10 = superior). The final score was derived by averaging the three competency scores (maximum 10 points) and scaling to 100.

The detailed scoring rubrics, containing full operational definitions and anchor points for all domains, are provided in Appendices 1, 2.

#### 2.2.4 Instructional satisfaction

Instructional satisfaction measured using a post-intervention questionnaire featuring 10 items rated on a 5-point Likert scale (1 = strongly disagree to 5 = strongly agree). This instrument evaluated students’ perceptions of learning efficiency, engagement, clinical reasoning enhancement, and overall course quality.

### 2.3 Statistical analysis

Statistical analyses were performed using SPSS Statistics (version 26.0, IBM Corp, United States) by a statistician blinded to group assignment. Continuous variables with normal distribution were compared using independent t-tests and presented as mean ± standard deviation. Non-normally distributed data were compared using Mann–Whitney U tests and presented as median (interquartile range) 3. Categorical variables were compared using Chi-square tests48 and presented as frequencies (%). A two-tailed *p*-value < 0.05 was considered statistically significant. Effect sizes were calculated using appropriate measures (Cohen’s d for parametric tests, rank-biserial correlation for non-parametric tests).

## 3 Results

### 3.1 Demographic data

**Figure 1:**
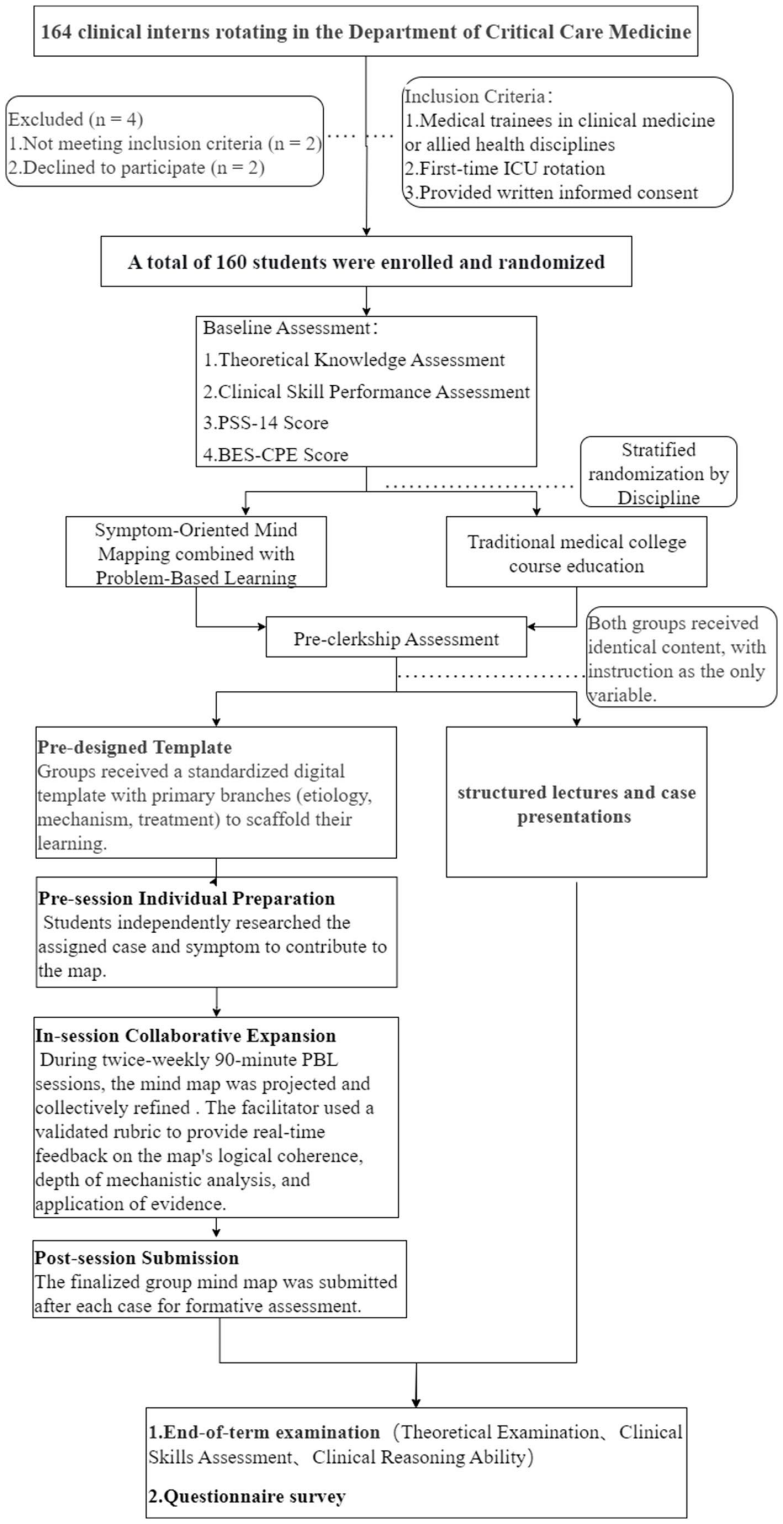
SOM-PBL strategy design teaching process flowchart.

This study enrolled 160 clinical clerkship trainees, with 80 participants each allocated to the SOM-PBL and control groups (Figure 1). Homogeneity analysis of baseline characteristics demonstrated no statistically significant differences in age, gender distribution, or pre-intervention theoretical performance scores between cohorts (*p* > 0.05), confirming effective randomization and establishing baseline comparability (all *p* > 0.05; Table 1).

**Table 1:** Comparison of general information between the two groups.

Characteristics		SOM-PBL	Control	χ^2^/t/z	*p* value
Sex	Male, n(%)	34(42.50)	40(50.00)	0.905	0.341
	Female, n(%)	46(57.50)	40(50.00)		
Age[year, M(*P*_25_, P_75_)]		22(22, 23)	22(22, 22)	−0.636	0.525
Medical Universities^a^, n(%)	a	7(8.75)	10(12.50)	0.643	0.886
	b	41(51.25)	38(47.50)		
	c	27(33.75)	27(33.75)		
	d	5(6.25)	5(6.25)		
Discipline^b^, n(%)	Clinical medicine	56(70.00)	51(63.75)	1.462	0.917
	Rehabilitation Therapy	5(6.25)	8(10.00)		
	Dentistry	2(2.50)	3(3.75)		
	Pharmacy	2(2.50)	3(3.75)		
	Medical Laboratory Technology	6(7.50)	7(8.75)		
	Medical Imaging Technology	9(11.25)	8(10.00)		
Previous semester theoretical course score [*M*(*P*_25_, P_75_)]^c^		94(92, 95)	93(91, 95)	−1.204	0.229
Previous semester clinical skill performance score [*M*(*P*_25_, P_75_)]^c^		89(85, 92)	89(87, 91.75)	−0.789	0.430
Perceived stress scale-14 items (PSS-14)^d^ [*M*(*P*_25_, P_75_)]		37(33, 40)	38(33.75, 40)	−0.405	0.685
Belongingness scale–clinical practice environment (BES-CPE)^e^ [*M*(*P*_25_, P_75_)]		106(102, 110)	106(100.50, 112)	−0.629	0.529
Clinical practicum engagement [%, M(*P*_25_, P_75_)]		99(95, 100)	99(95, 100)	−0.250	0.803

a a = Anhui University of Science and Technology; b = Jiangsu Vocational College of Health; c = Nanjing Medical University; d = Other institutions.

b The ‘Discipline’ category represents the students’ primary academic major prior to the clerkship.

c Scores are out of a maximum of 100 points.

d PSS-14, Perceived Stress Scale-14 items; a 14-item instrument measuring the degree to which situations in one’s life are appraised as stressful.

e Belongingness Scale--Clinical Practice Environment; a self-report instrument assessing students’ feelings of acceptance, value, and fit within the clinical learning environment.

### 3.2 Effectiveness evaluation

The SOM-PBL group demonstrated significantly higher theoretical examination scores compared to the control group (median [IQR]: 77 [73, 80] vs. 74 [72, 77]; *p* < 0.05), with a median difference of 3 points and a moderate effect size (Cohen’s d = 0.455). The distribution of scores shows a consistent upward shift across the interquartile range, with a notable 3-point difference at both the median and the 75th percentile (80 in SOM-PBL vs. 77 in controls), indicating a robust and generalized improvement in theoretical knowledge acquisition within the intervention group.

In contrast, no significant difference was observed in procedural skills scores between the two groups (median [IQR]: 92 [90, 94] for both groups; *p* = 0.938).

Additionally, the SOM-PBL cohort demonstrated superior clinical reasoning performance (median [IQR]: 88 [85, 93] vs. 87 [84, 89.75]; *p* < 0.05), corresponding to a one-point median difference and a moderate effect size (d = 0.43). Critically, the interquartile range (IQR) reveals a more pronounced separation at the 75th percentile (93 vs. 89.75), indicating that SOM-PBL was particularly effective in enhancing the clinical reasoning capabilities of the top quarter of students (Table 2).

**Table 2:** Comparison of end-of-term scores between the two groups.

Category	SOM-PBL	Control	Cohen’s d	*p* value
Theoretical Examination Score [*M*(*P*_25_, P_75_)]^a^	77(73, 80)	74(72, 77)	0.455	0.002
Clinical Skills Assessment Score [*M*(*P*_25_, P_75_)]^a^	92(90, 94)	92(90, 94)	0.029	0.938
Clinical Reasoning Ability Score [*M*(*P*_25_, P_75_)]^a^	88(85, 93)	87(84, 89.75)	0.428	0.012

a Scores are out of a maximum of 100 points.

Although a statistically significant difference was observed in pathophysiology recognition (*p* = 0.048), the identical median values (20 points) and small effect size (d = 0.323) indicate limited practical educational impact. The somewhat broader IQR in the SOM-PBL group (18.25–23 vs. 17–21.75) may reflect modestly more consistent performance among learners.

A moderate advantage was noted in monitoring technology application, with the SOM-PBL group scoring slightly higher [15 (13–16.75) vs. 14 (12–16); *p* = 0.025; d = 0.332], suggesting tentative improvement in practical knowledge application. In contrast, no significant difference was found in clinical decision-making (*p* = 0.506), indicating that short-term SOM-PBL did not improve therapeutic judgment compared to conventional training (Table 3).

**Table 3:** Comparison of theoretical knowledge acquisition between the two groups.

Category	SOM-PBL	Control	Cohen’s d	*p* value
Pathophysiology and Disease Recognition [*M*(*P*_25_, P_75_)]^a^	20(18.25, 23)	20(17, 21.75)	0.323	0.048
Monitoring Technology and Device Application [*M*(*P*_25_, P_75_)]^b^	15(13, 16.75)	14(12, 16)	0.332	0.025
Clinical Decision-Making [*M*(*P*_25_, P_75_)]^b^	13(11, 14)	12(11, 14)	0.074	0.506

a Scores are out of a maximum of 30 points.

b Scores are out of a maximum of 20 points.

In simulated clinical skills assessment, the SOM-PBL group demonstrated significantly higher scores in the management of complications and related decision-making (e.g., indications/contraindications) compared to the control group (*p* < 0.05). No significant differences were observed between groups in aseptic technique (*p* = 0.416) or core procedural protocol adherence (*p* = 0.136), though a trend toward improved performance in protocol execution was noted (Cohen’s d = 0.257; Table 4).

**Table 4:** Comparison of procedural skills performance between the two groups.

Category	SOM-PBL	Control	Cohen’s d	*p* value
Aseptic Principle [*M*(*P*_25_, P_75_)]^a^	7(6, 8)	7(6, 8)	0.154	0.416
Procedural Protocol [*M*(*P*_25_, P_75_)]^a^	7(6.5, 8)	7(6, 8)	0.257	0.136
Adverse Events [*M*(*P*_25_, P_75_)]^a^	7(7, 8)	7(6, 8)	0.437	0.012

a Scores are out of a maximum of 10 points.

In clinical reasoning assessments, the SOM-PBL group showed significantly higher performance in diagnostic cognitive rigor [8 (8–9) vs. 8 (7–8); *p* = 0.007; d = 0.447], reflecting improved logical analysis and hypothesis differentiation in complex cases. No substantial differences were found between groups in integrative clinical history analysis (*p* = 0.609) or time-critical decision-making (*p* = 0.910; Table 5).

**Table 5:** Comparison of clinical reasoning abilities between the two groups.

Category	SOM-PBL	Control	Cohen’s d	*p* value
Integrative Clinical History Analysis [*M*(*P*_25_, P_75_)]^a^	8(7, 8)	8(7, 8)	0.088	0.609
Diagnostic Cognitive Rigor [*M*(*P*_25_, P_75_)]^a^	8(8, 9)	8(7, 8)	0.447	0.007
Time-Critical Decision Determinants [*M*(*P*_25_, P_75_)]^a^	7(7, 8)	8(8, 9)	0.032	0.910

a Scores are out of a maximum of 10 points.

### 3.3 Questionnaire survey

**Figure 2:**
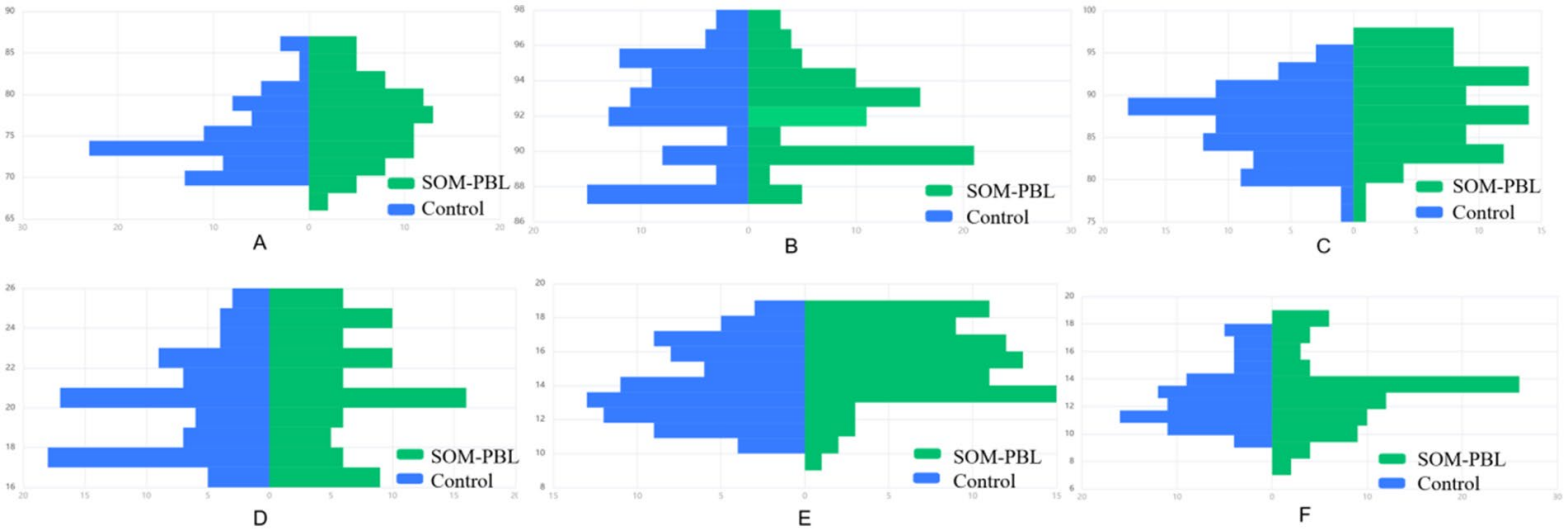
Comparative analysis of end-of-term assessment results between groups. **(A)** Theoretical examination scores, **(B)** clinical skills assessment scores, **(C)** clinical reasoning ability scores, **(D)** pathophysiology and disease recognition, **(E)** monitoring technology and device application, **(F)** clinical decision-making.

**Figure 3:**
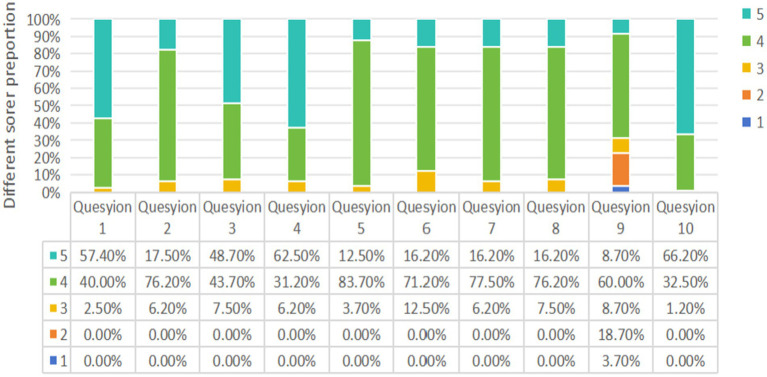
Results of questionnaire survey: the proportion of students’ ratings for each question. The survey adopts Likert five-level scoring method (1, strongly disagree; 2, disagree; 3, undecided; 4, agree; 5, strongly agree).

As depicted in Figure 2, survey results revealed that over 80% of students rated items 1 through 8 with scores of 4 or 5, indicating predominantly positive evaluations of the SOM-PBL (Symptom-Oriented combined with Problem-Based Learning) instructional approach across dimensions including learning efficiency, engagement, enhancement of clinical reasoning abilities, and overall course satisfaction. However, students also acknowledged certain limitations associated with this novel pedagogical model; specifically, for item 9, 25 students (31.1%) assigned scores ranging from 1 to 3, suggesting a perception among some participants that the SOM-PBL method contributed to an increased learning burden (Figure 3; Table 6).

**Table 6:** Questionnaire survey.

Evaluation content	1	2	3	4	5
Concise learning objectives facilitate comprehension of core curricular content	□	□	□	□	□
Enhanced learner engagement promotes rapid transition into learning readiness	□	□	□	□	□
Instructional methodology augments cognitive integration	□	□	□	□	□
Increased clinician confidence in clinical decision-making	□	□	□	□	□
Strengthened interprofessional collaborative competencies	□	□	□	□	□
Expanded opportunities for autonomous clinical practice	□	□	□	□	□
Elevated faculty engagement and interactive teaching dynamics	□	□	□	□	□
Contributed to professional identity formation	□	□	□	□	□
SOM-PBL implementation imposed additional cognitive load	□	□	□	□	□
High satisfaction with clinical learning environments, rotation scheduling, and didactic sessions	□	□	□	□	□

## 4 Discussion

Our randomized controlled trial demonstrated that the SOM-PBL model offers significant advantages over traditional teaching in the context of ICU clerkships. The intervention group showed superior performance in integrating theoretical knowledge and in clinical reasoning, as evidenced by significant improvements in key subdomains such as pathophysiology recognition and device application. Importantly, this benefit was most pronounced among students in the upper quartile of performance. However, SOM-PBL did not lead to better procedural skills, and it was associated with a perceived increase in cognitive load by a subset of learners.

The primary challenge in clinical clerkship teaching within intensive care medicine lies in cultivating complex knowledge integration and clinical decision-making under high-acuity conditions. Although traditional PBL improves clinical reasoning, students often struggle with knowledge fragmentation and unclear diagnostic pathways when managing multifactorial critical conditions. This study developed and evaluated a novel instructional model integrating Symptom-Oriented Mind Mapping with PBL—SOM-PBL—to scaffold cognitive structuring and clinical reasoning within a dual pedagogical framework. This integrated approach cultivates more systematic and accurate judgment in managing acute critical illnesses, strengthening students’ capacity for evidence-based, systematic decision-making in high-acuity contexts and effectively advancing the translation of clinical theory into practice (22).

This system-based perspective facilitates a comprehensive view of the pathophysiological alterations induced by the disease process, enabling students to demonstrate stronger capabilities in cross-disciplinary knowledge integration when addressing complex clinical problems (23). This aligns with cognitive schema theory, which emphasizes the role of structured knowledge architectures in clinical reasoning (24). Furthermore, the iterative PBL cycle—guiding students through data interpretation, hypothesis generation, evidence retrieval, and management planning—complemented the conceptual clarity provided by mind maps (25). This synergy likely contributed to the observed superiority of SOM-PBL in fostering systematic clinical decision-making (26). The fact that the biggest gain occurred among higher-performing students (those scoring at the 75th percentile) further suggests that SOM-PBL helps advanced learners excel even further. Unexpectedly, no significant intergroup differences were found in procedural skills. This may be attributed to the cognitive—rather than psychomotor—focus of the SOM-PBL intervention (27). Proficiency in technical skills often requires repetitive hands-on practice, which was not the emphasis of this model. This finding suggests that SOM-PBL should be combined with simulation-based training to achieve comprehensive clinical competency. We also noticed something interesting about cognitive load. About one-third of students reported that the SOM-PBL method felt more demanding. This might seem to contradict the idea that mind mapping simplifies learning—but actually, it reflects an important distinction in learning psychology. Some types of mental effort are productive: they lead to stronger and longer-lasting understanding. It is possible that the active, engaged style of SOM-PBL required more initial effort, which paid off in better knowledge integration and application (28). To address this, iterative refinements such as pre-constructed template scaffolds, collaborative digital mind-mapping, and just-in-time micro-lectures were incorporated to distribute cognitive effort and sustain engagement.

This study possesses several notable strengths. First, it introduces a novel, integrated SOM-PBL pedagogy that systematically combines symptom-oriented cognitive structuring with collaborative problem-based learning, offering a practical instructional strategy for complex clinical education. Second, the use of a randomized controlled design with stratified allocation by academic major enhances internal validity and minimizes confounding due to pre-existing knowledge differences. Third, outcome assessment was robust, incorporating multiple dimensions—theoretical knowledge, practical skills, clinical reasoning, and learner satisfaction—evaluated using standardized instruments and blinded assessors, thereby increasing the reliability and comprehensiveness of the findings. Finally, the study was conducted under real-world clinical training conditions, which supports the ecological validity and potential scalability of the SOM-PBL model in similar medical education environments.

This study has several limitations. First, the single-center design may limit the generalizability of our findings to institutions with different educational structures or resources. Although the real-world setting of our trial may support ecological validity within comparable contexts, we caution against overgeneralization, as medical education systems vary substantially across countries and institutions. Broader scalability must be demonstrated through multi-center studies.

Second, the inability to blind participants and instructors may introduce performance bias, and potential cross-group contamination could attenuate the measured intervention effect. Third, the brief 4-week intervention period prevents assessment of long-term knowledge retention.

Furthermore, despite randomization, residual confounding from unmeasured variables such as individual motivation or prior study time cannot be excluded. Some procedural assessments may also have been constrained by near-ceiling effects. Finally, although stratified randomization balanced the diverse academic majors included in this study, residual heterogeneity in pre-clerkship backgrounds remains a potential source of variation. Our study was not powered to conduct subgroup analyses across disciplines, and differential efficacy of the intervention among various student populations remains an important area for future investigation.

In conclusion, the SOM-PBL model effectively supports knowledge integration and clinical reasoning in critical care education by combining visual knowledge structuring with iterative case-based reasoning. It represents a promising strategy for bridging the gap between theoretical knowledge and clinical practice, particularly in complex, high-stakes learning environments.

## 5 Conclusion

The SOM-PBL model significantly enhances knowledge integration and clinical decision-making in critical care trainees, effectively bridging knowledge fragmentation and the theory-practice gap inherent in traditional pedagogy. Its efficacy establishes a standardized operational paradigm for critical care education with considerable implementation potential. Future research will implement multicenter, large-sample randomized controlled trials involving trainee cohorts from diverse hospital tiers to comprehensively evaluate the generalizability and accessibility of this instructional framework.

## Data Availability

The raw data supporting the conclusions of this article will be made available by the authors, without undue reservation.
