# Supplementary material for: The effectiveness of symptom-oriented mind mapping combined with problem-based learning in critical care clerkships: a randomized controlled trial
Source: Front Public Health. 2025 Oct 17;13:1682687. doi: 10.3389/fpubh.2025.1682687 (PMC12575364; doi:10.3389/fpubh.2025.1682687)
Supplement: Supplementary file 1 [file Table_1.docx]

**Appendix 1:**

**Procedural Skills Assessment Rubric**

Scoring scale: 0-10 points for each domain (Maximum total: 30 points, scaled to 100)

| **Domain 1: Aseptic Technique** | | |
| --- | --- | --- |
| **Score Range** | **Performance Level** | **Description** |
| 9-10 | Excellent | Performs flawless hand hygiene; dons sterile gloves and gown without contamination; maintains impeccable sterile field throughout; handles all sterile supplies correctly |
| 7-8 | Proficient | Minor, isolated break in technique with immediate self-correction; sterile field largely maintained |
| 5-6 | Competent | Multiple minor breaks in technique but no major contamination; requires occasional prompting |
| 0-4 | Needs Improvement | Major break in sterile technique (e.g., touches non-sterile area); demonstrates lack of understanding of principles |
| **Domain 2: Procedural Protocol** | | |
| **Score Range** | **Performance Level** | **Description** |
| 9-10 | Excellent | Completes all pre-procedural preparations flawlessly; executes procedure with logical sequencing and exceptional fluency; demonstrates mastery in post-procedure management |
| 7-8 | Proficient | Completes most pre-procedural preparations; executes procedure with proper sequencing and adequate fluency; appropriate post-procedure management |
| 5-6 | Competent | Completes basic pre-procedural preparations; executes procedure with minor deviations in sequencing; basic post-procedure management |
| 0-4 | Needs Improvement | Incomplete pre-procedural preparations; significant errors in procedural sequencing; inadequate post-procedure management |
| **Domain 3: Management of Adverse Events** | | |
| **Score Range** | **Performance Level** | **Description** |
| 9-10 | Excellent | Anticipates potential complications; demonstrates exceptional skill in managing adverse events; takes appropriate preventive measures |
| 7-8 | Proficient | Recognizes and responds appropriately to adverse events; manages complications effectively |
| 5-6 | Competent | Recognizes obvious adverse events but response may be delayed or partially effective |
| 0-4 | Needs Improvement | Fails to recognize or inappropriately manages adverse events; unable to implement corrective actions |
