# Supplementary material for: The effectiveness of symptom-oriented mind mapping combined with problem-based learning in critical care clerkships: a randomized controlled trial
Source: Front Public Health. 2025 Oct 17;13:1682687. doi: 10.3389/fpubh.2025.1682687 (PMC12575364; doi:10.3389/fpubh.2025.1682687)
Supplement: Supplementary file 2 [file Table_2.docx]

**Appendix 2:**

**Clinical Reasoning Assessment Rubric**

**(Modified Mini-CEX)**

Scoring scale: 1-10 points for each competency (Maximum average: 10 points, scaled to 100)

| **Competency 1: Integrative Clinical History Analysis** | | |
| --- | --- | --- |
| **Score Range** | **Performance Level** | **Description** |
| 9-10 | Superior | Creates a compelling, insightful, and perfectly logical narrative that efficiently guides diagnostic reasoning |
| 7-8 | Good | Synthesizes information into a coherent story; timeline is mostly accurate and logical |
| 4-6 | Satisfactory | Gathers most relevant information but presentation is list-like rather than integrative; timeline may have gaps |
| 1-3 | Unsatisfactory | History is incomplete, fragmented, or illogical; fails to identify key information |
| **Competency 2: Diagnostic Cognitive Rigor** | | |
| **Score Range** | **Performance Level** | **Description** |
| 9-10 | Superior | Demonstrates exceptional critical thinking; considers comprehensive differential diagnoses with clear logical sequencing and prioritization |
| 7-8 | Good | Shows good analytical skills; considers multiple diagnostic hypotheses with reasonable prioritization |
| 4-6 | Satisfactory | Demonstrates basic analytical skills; considers limited differential diagnoses with some logical reasoning |
| 1-3 | Unsatisfactory | Shows minimal analytical thinking; limited or no differential diagnoses; relies on pattern recognition or guessing |
| **Competency 3: Time-Critical Decision Determinants** | | |
| **Score Range** | **Performance Level** | **Description** |
| 9-10 | Superior | Rapidly identifies life-threatening conditions; demonstrates exceptional decisiveness in initiating appropriate interventions; prioritizes actions perfectly based on acuity |
| 7-8 | Good | Recognizes critical conditions; makes timely decisions regarding interventions; generally appropriate prioritization |
| 4-6 | Satisfactory | Identifies obvious critical conditions but may be delayed; decisions may be hesitant or partially appropriate |
| 1-3 | Unsatisfactory | Fails to recognize critical conditions; significant delays in decision-making; inappropriate or dangerous prioritization |
